# Acanthamoeba castellanii Genotype T4 Stimulates the Production of Interleukin-10 as Well as Proinflammatory Cytokines in THP-1 Cells, Human Peripheral Blood Mononuclear Cells, and Human Monocyte-Derived Macrophages

**DOI:** 10.1128/IAI.00345-16

**Published:** 2016-09-19

**Authors:** Antonella Mattana, Manuela Sanna, Antonella Cano, Giuseppe Delogu, Giuseppe Erre, Craig W. Roberts, Fiona L. Henriquez, Pier Luigi Fiori, Piero Cappuccinelli

**Affiliations:** a Department of Biomedical Sciences, University of Sassari, Sassari, Italy; b Strathclyde Institute of Pharmacy & Biomedical Sciences, University of Strathclyde, Glasgow, United Kingdom; c IBEHR, School of Science & Sport, University of the West of Scotland, Paisley, United Kingdom; Cornell University

## Abstract

Free-living amoebae of the genus Acanthamoeba can cause severe and chronic infections in humans, mainly localized in immune privileged sites, such as the brain and the eye. Monocytes/macrophages are thought to be involved in Acanthamoeba infections, but little is known about how these facultative parasites influence their functions. The aim of this work was to investigate the effects of Acanthamoeba on human monocytes/macrophages during the early phase of infection. Here, THP-1 cells, primary human monocytes isolated from peripheral blood, and human monocyte-derived macrophages were either coincubated with trophozoites of a clinical isolate of Acanthamoeba (genotype T4) or stimulated with amoeba-derived cell-free conditioned medium. Production of proinflammatory cytokines (tumor necrosis factor alpha [TNF-α], interleukin-6 [IL-6], and IL-12), anti-inflammatory cytokine (IL-10), and chemokine (IL-8) was evaluated at specific hours poststimulation (ranging from 1.5 h to 23 h). We showed that both Acanthamoeba trophozoites and soluble amoebic products induce an early anti-inflammatory monocyte-macrophage phenotype, characterized by significant production of IL-10; furthermore, challenge with either trophozoites or their soluble metabolites stimulate both proinflammatory cytokines and chemokine production, suggesting that this protozoan infection results from the early induction of coexisting, opposed immune responses. Results reported in this paper confirm that the production of proinflammatory cytokines and chemokines by monocytes and macrophages can play a role in the development of the inflammatory response during Acanthamoeba infections. Furthermore, we demonstrate for the first time that Acanthamoeba stimulates IL-10 production in human innate immune cells, which might both promote the immune evasion of Acanthamoeba and limit the induced inflammatory response.

## INTRODUCTION

Ubiquitous amoebae of the genus Acanthamoeba are amphizoic protozoa with the ability to exist both as free-living organisms in nature and as parasites within host tissues (1). Numerous reports indicate that several species of Acanthamoeba can infect humans, producing severe ocular disease and almost invariably fatal neurological disease (2 – 7). Human infections are generally site specific: corneal and neural tissues are the primary targets, although other tissues can be affected. Acanthamoeba can act as opportunistic as well as nonopportunistic pathogens.

In immunocompromised patients, Acanthamoeba cause an intense, progressive, chronic, often fatal granulomatous amoebic meningoencephalitis, GAE, as well as lesions within eye, skin, lungs, and bones. More recently, GAE also has been reported in healthy individuals (3, 8 – 11). In some cases, these apparently immunocompetent GAE patients had a history of exposed skin lesions, chronic sinusitis, rhinitis, or pneumonia (12), suggesting that Acanthamoeba can reach the central nervous system through three main portals of entry: skin lesions, the respiratory tract, and the olfactory neuroepithelium.

In immunocompetent individuals, Acanthamoeba are agents of a painful, progressive, sight-threatening corneal infection, termed Acanthamoeba keratitis (AK). The corneal epithelium is primarily affected with evident inflammation; however, infection can progress within the deeper layers and, if not promptly diagnosed, can lead to partial or complete loss of vision. AK is mainly associated with use of contact lenses (CLs). This is for a number of reasons, including the ability of CLs to cause microcorneal trauma, and their inappropriate use, maintenance, and cleaning can facilitate their contamination with Acanthamoeba (13).

Understanding the pathogenesis of disease, including the complexity of the immunological mechanisms involved, is essential for the development of effective prophylactic and therapeutic strategies. Host resistance mechanisms operative against Acanthamoeba may involve innate and acquired immunity. The initial innate immune response and the formation of granulomas in the brain and other tissues are presumed to be essential events for impeding the invasion and dissemination of Acanthamoeba trophozoites (14).

Many studies have indicated that macrophages are important effectors in the granulomatous reaction surrounding infected tissues (15). In addition, it has been reported that the elimination of conjunctival-corneal macrophages *in vivo* results in a severe prolonged course of Acanthamoeba keratitis without resolution (16). A study on amoeba-macrophage interactions has also established that activated macrophages can injure weakly pathogenic species of Acanthamoeba, whereas highly pathogenic species can evade the microbicidal activity of these effector cells (17). Nevertheless, very little is known about the biochemical mechanisms that occur in human monocytes upon exposure to Acanthamoeba trophozoites or their products.

Cells of the monocyte/macrophage lineage can modulate the immune response to microbes and microbial products by both the induction of membrane-associated signaling molecules and the synthesis and secretion of soluble cytokines. Upon microbial recognition, activated monocytes/macrophages can release a wide range of proinflammatory cytokines, such as tumor necrosis factor alpha (TNF-α), interleukins (IL-1, IL-6, IL-12, and IL-18), and chemokines (IL-8), that amplify the innate and adaptive immune responses leading to pathogen clearance and resolution of inflammation.

Innate immune cells also can release a number of cytokines with the capacity to inhibit functions of effector cells and suppress inflammation. IL-10 is probably the most important anti-inflammatory cytokine for limiting the overwhelming immune reactions that might lead to tissue injury. Mononuclear phagocytes and lymphocytes represent the main source of IL-10. It is known that IL-10 inhibits the production of proinflammatory cytokines and chemokines and reduces the antigen-presenting capacity of antigen-presenting cells (dendritic cells) by downregulating major histocompatibility complex class II (MHC-II) molecules (18).

On the basis of all this knowledge, the present investigation was undertaken to determine the mechanisms that occur, during the early phase of infection, between human monocytes/macrophages and Acanthamoeba.

To monitor monocyte/macrophage activation, we focused on the cytokine production by human monocytes isolated from peripheral blood (PBMCs) and human monocyte-derived macrophages (MDMs). THP-1 cells have been used to perform a large number of experiments, limiting the need for human blood donors.

The present study determines the kinetics of TNF-α, IL-6, IL-8, IL-10, and IL-12 secretion by THP-1 cells (19), PBMCs, and MDMs in response to Acanthamoeba trophozoites and soluble products contained in amoebic conditioned cell-free medium (aCM). We demonstrate that Acanthamoeba stimulates proinflammatory cytokine release as well as the early production of IL-10 in these innate immune cells.

## MATERIALS AND METHODS

### Reagents and materials.

RPMI 1640 medium and fetal calf serum (FCS) were obtained from Gibco-BRL/Life Technologies, Italy. HEPES buffer, penicillin G-streptomycin sulfate, Dulbecco's phosphate-buffered saline solution (PBS), Histopaque 1077, and Escherichia coli lipopolysaccharide (LPS) were obtained from Sigma-Aldrich Srl, Milan, Italy. The monoclonal antibody Leu-M3 (anti-CD14) was from Becton Dickinson (San Jose, CA). Enzyme-linked immunosorbent assay (ELISA) kits (human TNF-α DuoSet, human IL-6 DuoSet, human IL-12 p70 DuoSet, human CXCL8/IL-8 DuoSet, and human IL-10 DuoSet) were obtained from R&D Systems (Space Srl, Milan, Italy).

### Amoeba culture conditions.

Our study was performed using trophozoites of A. castellanii genotype T4 (20, 21), isolated from the corneal ulcer of a soft contact lens wearer (in Ancona, Italy) and axenically grown at 25°C in proteose-yeast extract-glucose (PYG) medium (22). Species and genotype identification of this isolate were based on cyst morphology, PCR analysis using primers JDP1 and JDP2 (specific for 18S rRNA gene stretch ASA.S1), and DNA sequencing (23). It is known that Acanthamoeba can be reservoirs for amoeba-resistant microorganisms such as bacteria, fungi, and viruses (24 – 26). For this reason, PCR with universal bacterial primers was applied in order to verify the absence of bacteria within the trophozoites used in the experiments (27), and they were free of any contaminants. Our previous observations showed that this A. castellanii isolate was able to damage the human amnion-derived epithelial cell line (Wish) by both contact-dependent and contact-independent mechanisms (22). Amoebae used for the inocula were taken from logarithmic-phase cultures. In each experiment cell viability was >95%, as determined by the nigrosin dye exclusion method.

### Preparation of aCM.

Amoeba cell-free conditioned medium (aCM) was prepared as previously described (20, 22, 28, 29). Briefly, amoebae were washed twice in PBS (2.7 mM KCl,1.5 mM KH_2_PO_4_, 136.9 mM NaCl, 8.9 mM Na_2_HPO_4_ · 7H_2_O, pH 7.4), suspended (4 × 10^6^ cell/ml) in RPMI 1640 medium containing 20 mM HEPES, and finally incubated for 2 h at 25°C. Amoeba cell-free supernatant was obtained by centrifugation at 500 × *g* for 15 min and used as conditioned medium (aCM) after the addition of 5% heat-inactivated FCS. For each experiment, aCM was used immediately after processing.

### Culture conditions of THP-1 cell line.

THP-1 cells (Sigma-Aldrich, Milan, Italy) were maintained in continuous culture in RPMI 1640 medium supplemented with 10% heat-inactivated FCS, 100 U of penicillin G, and 100 μg/ml of streptomycin sulfate and grown in 25-cm^2^ sterile plastic flasks at 37°C in a humid atmosphere containing 5% CO_2_. In each experiment cell viability was >95%, as determined by the nigrosin dye exclusion method.

### Human monocyte isolation and MDM differentiation.

Human venous whole blood was obtained from three different healthy volunteers at the Hospital-University Company (AUO-SS, Sassari, Italy), where all of the relevant ethical reviews and approvals were granted for collection and manipulation at the time. Donors had taken no medication for at least 2 weeks before they donated blood. Monocytes were isolated from aliquots of blood (40 ml) as previously described (30). Briefly, blood was diluted with phosphate-buffered saline (pH 7.4), layered over a Histopaque (density, 1.077 g/cm^3^) gradient solution, centrifuged (400 × *g*, 25 min, room temperature), and recovered by thin suction at the interface. The mononuclear cell layer was washed twice by centrifuging with PBS (200 × *g*, 10 min, room temperature) and then suspended in RPMI 1640 medium supplemented with 20% heat-inactivated FCS, 2 mM glutamine, 10 mM HEPES, and antibiotics. Purified PBMCs were obtained by cell adhesion in 25-cm^2^ sterile plastic flasks; after 22 h of incubation at 37°C in 5% CO_2_, nonadherent cells (mainly lymphocytes) were removed by gentle washes with PBS. Adherent cells were characterized by immunofluorescence with the monoclonal antibody LEUm3 (anti-CD14), a marker for the LPS receptor, that was used to define blood monocytes (31). MDMs were prepared from monocytes cultured for 8 to 10 days in a 5% CO_2_ incubator at 37°C in RPMI 1640 medium containing 20% FCS, 2 mM glutamine, 10 mM HEPES, and antibiotics; medium was changed every 2 to 3 days. MDMs at day 9 were characterized by evaluating the decrease in the surface monocyte marker CD14. PBMCs and MDMs obtained from each donor were used separately. In each experiment cell viability was >95%, as determined by the nigrosin dye exclusion test.

### Experimental design.

Preliminary tests were performed to optimize the number of target cells (THP-1 cells, human PBMCs, and MDMs) and trophozoites and the concentration of LPS to apply in the experiments.

### Target cell stimulation for the analysis of Acanthamoeba-induced cytokine production.

For each experiment, 0.2 ml of target cell suspension (2 × 10^5^ cells) was seeded in 24-well tissue culture plates. Subsequently, 0.8 ml of trophozoite suspension (2.5 × 10^4^ trophozoites/ml) in RPMI medium–20 mM HEPES supplemented with 5% FCS (complete RPMI medium) was added to the wells for the coincubation trophozoite/target cell condition (amoeba/target cell ratio, 1:10), while the stimulation with amoebic conditioned cell-free medium was performed by adding 0.8 ml of aCM (obtained as described above). Target cells were also incubated with complete RPMI medium containing E. coli LPS (final concentration of 10 ng/ml) as a positive control. Complete RPMI medium (0.8 ml) was added to the wells as a negative-control condition. Plates were incubated at 37°C in the presence of 5% CO_2_ throughout the duration of the experiment. At time intervals ranging from 1.5 to 23 h, plates were centrifuged (200 × *g* for 10 min), and culture supernatants were collected and stored at −80°C until cytokine production evaluation. Cytokine concentrations in THP-1 experimental supernatants were evaluated at 1.5, 4, 6, and 23 h poststimulation, whereas for both PBMCs and MDMs culture supernatants were evaluated at 1.5, 3, 5, and 18 h poststimulation.

### Measurement of cytokine concentrations by ELISA.

The concentration of each examined cytokine (TNF-α, IL-6, IL-8, IL-10, and IL-12) was measured quantitatively with the use of the relevant kit of the DuoSet ELISA development system (R&D Systems) according to the manufacturer's instructions. Optical density was measured using a spectrophotometer (Servamax) at a 450-nm wavelength. Cytokine concentration was calculated, based on the values of a standard curve, using the software SoftMax Pro 5.0.1. Concentrations were expressed in picograms per milliliter.

### Statistical analyses.

All experiments were performed in triplicate and were repeated at least three times. Data are presented as the means ± standard errors of the means (SEM). Statistical difference was evaluated with the two-tailed Student *t* test and one-way analysis of variance (ANOVA) followed by Dunnett's multiple-comparison test using GraphPad Prism, version 5 (GraphPad Software Inc.). *P* values of <0.05 and <0.01 were considered significant and very significant, respectively.

## RESULTS

### PBMC and MDM characterization.

The great majority of adherent cells (93% ± 5%, *n* = 3) used in our experiments as PBMCs were CD14^+^, while a significantly lower percentage of MDMs (8% ± 0.95%, *n* = 3) were CD14^+^ at day 9.

### A. castellanii (genotype T4) modulates the production of proinflammatory cytokines (TNF-α, IL-6, and IL-12) in human THP-1 cells, monocytes, and macrophages in a time-dependent manner. (i) TNF-α.

**FIG 1:**
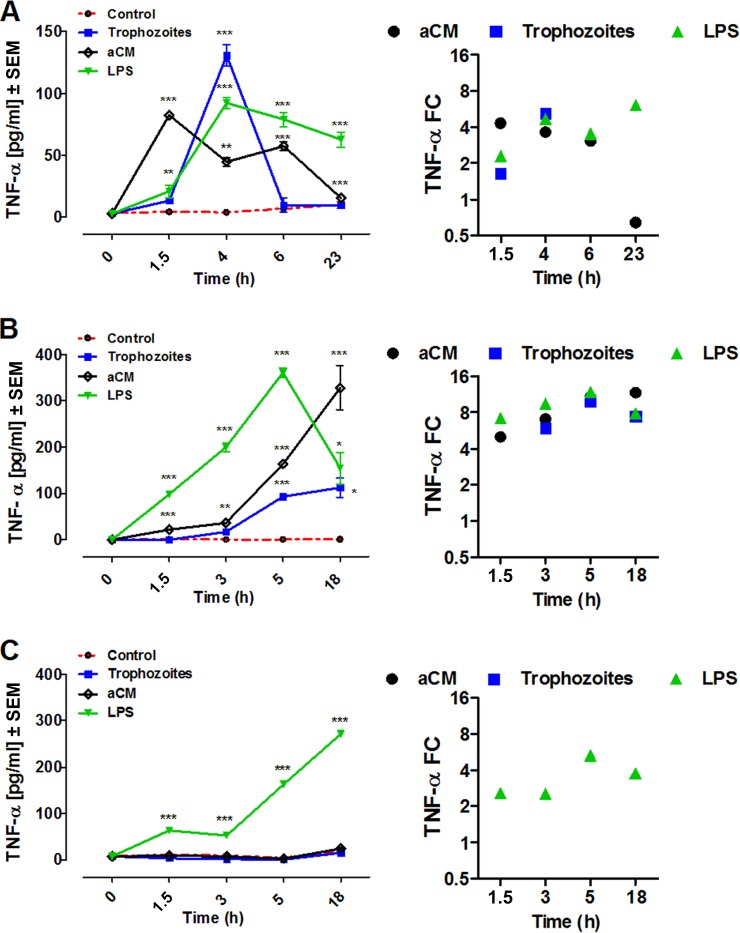
TNF-α production by THP-1 (A), PBMCs (B), and MDMs (C) after coincubation with trophozoites of A. castellanii (trophozoites) or stimulation with aCM. A total of 2 × 10^5^ of either target cells were challenged with either 2 × 10^4^ trophozoites at a ratio of 10:1 or with 800 μl of aCM (final dilution, 4:5 in the culture medium). TNF-α concentration in THP-1 experimental supernatants was evaluated at 1.5, 4, 6, and 23 h poststimulation, whereas in both PBMC and MDM experimental supernatants it was evaluated at 1.5, 3, 5, and 18 h poststimulation. All three cellular models were stimulated with 10 ng/ml LPS as a positive control (LPS), whereas unstimulated cells (control) were considered the negative control. Experiments were performed in triplicate and repeated at least 3 times. Results represent the mean values ± SEM of either *n* = 5 (THP-1) or *n* = 3 (PBMCs and MDMs) samples. One-way ANOVA was applied for each time point. Dunnett's multiple-comparison test was performed to evaluate differences between the experimental condition means and the control at each time point. In the graphs, significance between the experimental conditions and the control are indicated as *P* values of <0.05 (*), <0.005 (**), and <0.0001 (***). On the right, the same results are expressed as fold change (FC) over the unstimulated cells.

At 1.5 h postinfection, significant TNF-α production was detected in THP-1 cultures stimulated with aCM (82.55 ± 0.8776 pg/ml, *n* = 5, *P* < 0.0001) and LPS (20.88 ± 5.144 pg/ml, *n* = 5, *P* < 0.005) compared with noninfected controls, showing significant levels throughout the course of the stimulation. Of interest, aCM elicited a biphasic release curve, characterized first by a rapid increase at 1.5 h and then a second peak (57.1433 ± 3.681 pg/ml, *n* = 5, *P* < 0.0001) at 6 h (Fig. 1A). Coincubation of THP-1 with trophozoites caused a significant increase of TNF-α, which peaked at 4 h (130.747 ± 8.689 pg/ml, *n* = 5, *P* < 0.0001) and then decreased drastically (Fig. 1A). A TNF-α concentration of <10 pg/ml was detected in control THP-1 cells at all times (Fig. 1A).

TNF-α production in human PBMCs was significantly induced by aCM throughout the time course, in particular peaking at 18 h poststimulation (328.521 ± 47.84 pg/ml, *n* = 3, *P* < 0.0001) (Fig. 1B). On the contrary, coincubation of human PBMCs with trophozoites showed a significant increase of TNF-α only after 3 h of coincubation, reaching the highest value at 5 h (93.66 ± 2.993 pg/ml, *n* = 3, *P* < 0.0001) and subsequently remaining constant (Fig. 1B).

A basal TNF-α concentration of <25 pg/ml was detected in nonstimulated human MDMs and in MDM cultures either stimulated with aCM or coincubated with trophozoites at any time. In contrast, the treatment with LPS caused significant production of TNF-α by these cells, peaking at 18 h poststimulation (Fig. 1C).

### (ii) IL-6.

**FIG 2:**
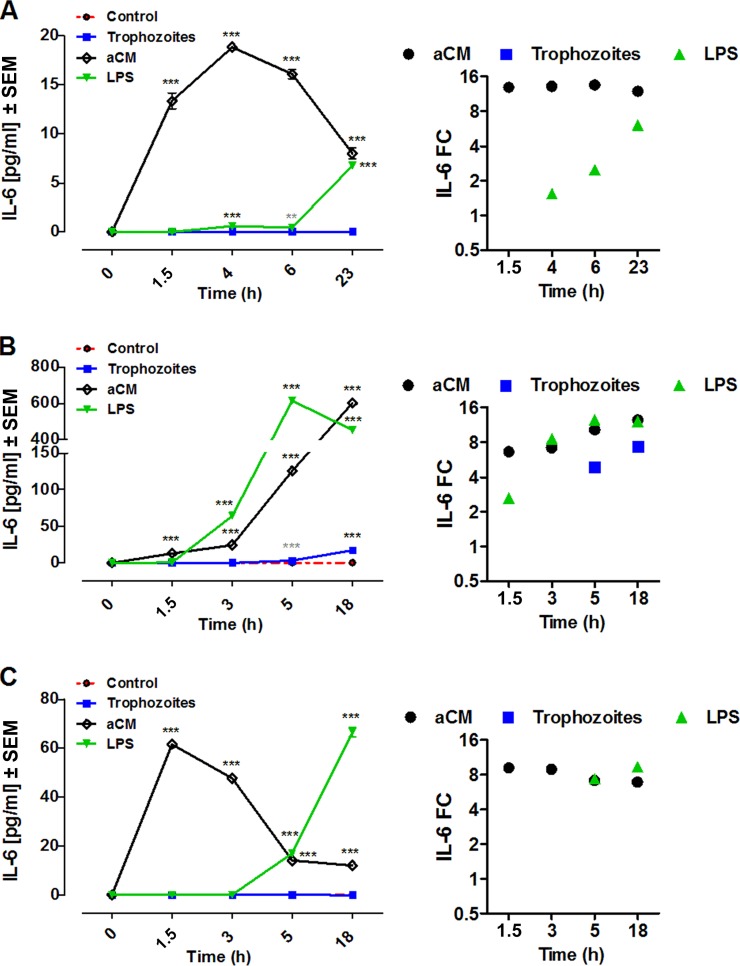
IL-6 production by THP-1 (A), PBMCs (B), and MDMs (C) after coincubation with trophozoites of A. castellanii (trophozoites) or stimulation with aCM. A total of 2 × 10^5^ of either target cells were challenged with either 2 × 10^4^ trophozoites, ratio of 10:1, or with 800 μl of aCM (final dilution of 4:5 in the culture medium). IL-6 concentration in THP-1 experimental supernatants was evaluated at 1.5, 4, 6, and 23 h poststimulation, whereas in both PBMC and MDM experimental supernatants it was evaluated at 1.5, 3, 5, and 18 h poststimulation. All three cellular models were stimulated with 10 ng/ml LPS as a positive control, whereas unstimulated cells (control) were considered the negative control. Experiments were performed in triplicate and repeated at least 3 times. Results represent the mean values ± SEM of either *n* = 5 (THP-1) or *n* = 3 (PBMCs and MDMs) samples. One-way ANOVA was applied for each time point. Dunnett's multiple-comparison test was performed to evaluate differences between the experimental condition means and the control at each time point. In the graphs, significances between the experimental conditions and the control are indicated as a *P* value of <0.0001 (***). Student *t* test was also applied at each time point to verify the significance of single pairs of means (single experimental condition versus control). In the graphs, significance between the experimental condition and the control, obtained with Student *t* test, are indicated as *P* values of <0.005 (**) and <0.0001 (***). On the right, the same results are expressed as fold change (FC) over the unstimulated cells.

aCM-stimulated THP-1 produced IL-6 throughout the course of the stimulation. In particular, IL-6 was significantly released after 1.5 h poststimulation (13.354 ± 0.796 pg/ml, *n* = 5, *P* < 0.0001), peaking at 4 h (18.85 ± 0.03395 pg/ml, *n* = 5, *P* < 0.0001) and subsequently decreasing until 23 h, still showing significant concentration values (8.000 ± 0.5774 pg/ml, *n* = 5, *P* < 0.0001). In contrast, coincubation with trophozoites did not induce release of IL-6 by THP-1. LPS-induced IL-6 production reached higher values after 23 h poststimulation (6.773 ± 0.01368 pg/ml, *n* = 5, *P* < 0.0001). Interestingly, the dynamics and profile of aCM-induced IL-6 production in THP-1 cells was quite dissimilar from that induced by LPS stimulation. In fact, aCM caused a significantly more rapid IL-6 production than LPS that peaked at 23 h poststimulation. IL-6 was not detectable in control THP-1 culture supernatants at any time (Fig. 2A).

PBMC stimulation with aCM induced a significant time-dependent release of IL-6 throughout the time course, reaching the highest value at 18 h poststimulation (602.0 ± 8.145 pg/ml, *n* = 3, *P* < 0.0001) (Fig. 2B). LPS-induced IL-6 production in this cellular model peaked at 5 h poststimulation (615.0 ± 13.28 pg/ml, *n* = 3, *P* < 0.0001) (Fig. 2B). Human PBMCs coincubated with trophozoites produced an increase of IL-6 from 5 h to 18 h postinfection (17.1612 ± 0.9115 pg/ml, *n* = 3), and this release was significant compared to the control (0.1 ± 0.05774 pg/ml, *n* = 3) only through the evaluation by Student *t* test (*P* < 0.0001) (Fig. 2B).

Human MDMs coincubated with trophozoites, as well as control MDM cultures, did not release IL-6, whereas stimulation with aCM induced a significant increase of IL-6 concentration during the time course with a pattern similar to that seen in THP-1 cells, characterized by a rapid and early peak at 1.5 h (61.56 ± 0.7817 pg/ml, *n* = 3, *P* < 0.0001), followed by a slow and gradual reduction. On the contrary, LPS peaked at 18 h poststimulation (66.56 ± 2.105 pg/ml, *n* = 3, *P* < 0.0001) (Fig. 2C).

### (iii) IL-12.

**FIG 3:**
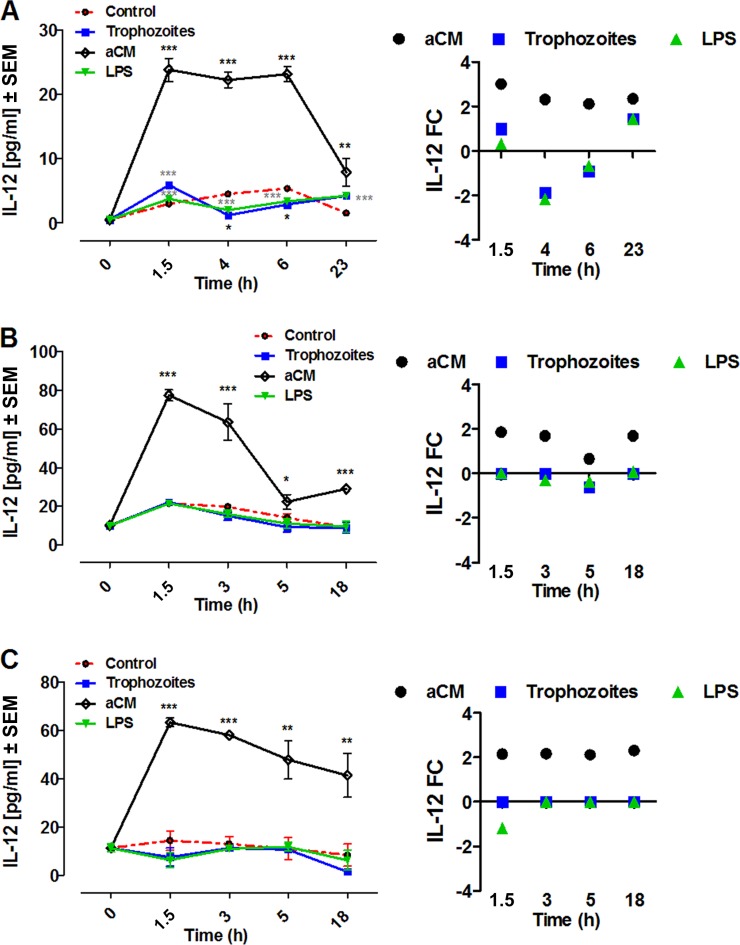
IL-12 production by THP-1 (A), PBMCs (B), and MDMs (C) after coincubation with trophozoites of A. castellanii (trophozoites) or stimulation with aCM. A total of 2 × 10^5^ of either target cells were challenged with either 2 × 10^4^ trophozoites, ratio of 10:1, or with 800 μl of aCM (final dilution of 4:5 in the culture medium). IL-12 concentration in THP-1 experimental supernatants was evaluated at 1.5, 4, 6, and 23 h poststimulation, whereas in both PBMCs and MDMs experimental supernatants it was evaluated at 1.5, 3, 5, and 18 h poststimulation. All three cellular models were stimulated with 10 ng/ml LPS as a positive control, whereas unstimulated cells (control) were considered the negative control. Experiments were performed in triplicate and repeated at least 3 times. Results represent the mean values ± SEM of either *n* = 5 (THP-1) or *n* = 3 (PBMCs and MDMs) samples. One-way ANOVA was applied for each time points. Dunnett's multiple-comparison test was performed to evaluate differences between the experimental condition means and the control at each time point. In the graphs, significance between the experimental conditions and the control are indicated as *P* values of <0.05 (*), <0.005 (**), and <0.0001 (***). Student *t* test was also applied at each time point to verify the significance of single pairs of means (single experimental condition versus control). In the graphs, significances between the experimental condition and the control, obtained with Student *t* test, are indicated as a *P* value of <0.0001 (***). On the right, the same results are expressed as fold change (FC) over the unstimulated cells.

IL-12 production increased progressively throughout the course of the experiment in aCM-stimulated THP-1 cells, human PBMCs, and MDMs, reaching the highest concentrations at 1.5 h postinfection compared with the respective control cultures. The IL-12 concentrations detected in THP-1 cells (23.78 ± 1.810 pg/ml, *n* = 5, *P* < 0.0001) were lower than those measured in PBMCs (77.44 ± 2.782 pg/ml, *n* = 3, *P* < 0.0001) and MDMs (63.34 ± 1.744 pg/ml, *n* = 3, *P* < 0.0001) (Fig. 3).

Coincubation with Acanthamoeba trophozoites did not induce IL-12 production by PBMCs and MDMs. Of interest, in the THP-1 culture a significant increase of IL-12 concentration was detected compared to the negative control (5.883 ± 0.001333, *n* = 5, *P* < 0.0001 by Student *t* test) at 1.5 h postcoincubation with trophozoites, followed by a significant decrease at 4 h (1.220 ± 0.00014 pg/ml, *n* = 5, *P* < 0.05) and 6 h (2.853 ± 0.0013 pg/ml, *n* = 5, *P* < 0.05) postcoincubation (Fig. 3A).

LPS stimulation did not induce any significant effect in the release of IL-12 by PBMCs and MDMs, whereas in THP-1 cells we observed a pattern similar to that induced by trophozoites (Fig. 3).

### A. castellanii (genotype T4) modulated the production of inflammatory chemokine IL-8 in human THP-1 cells, monocytes, and macrophages in a time-dependent manner.

**FIG 4:**
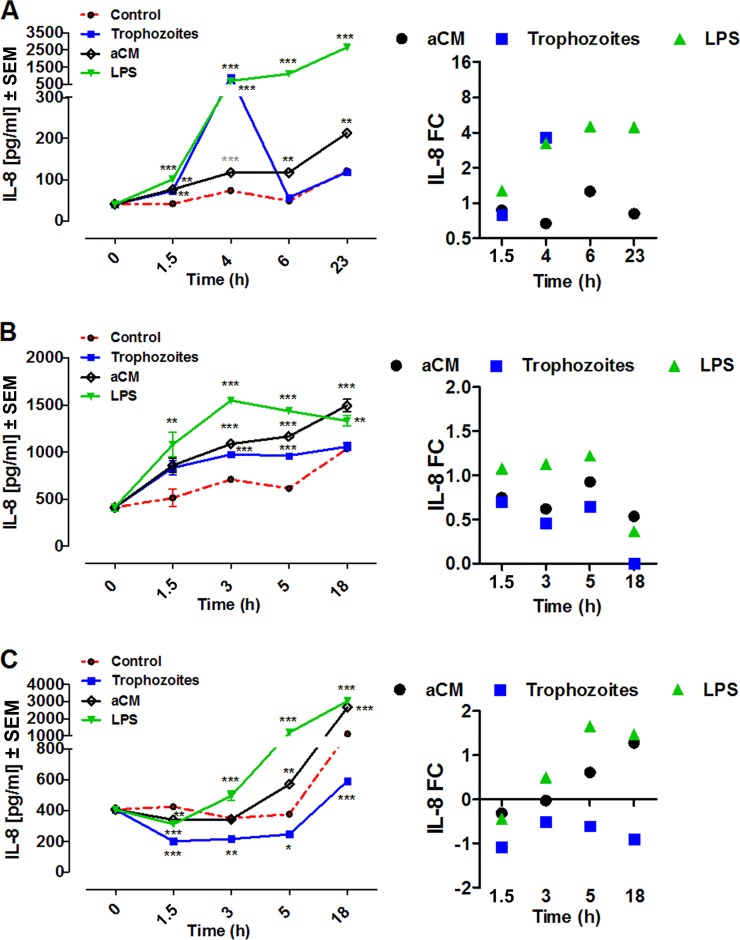
IL-8 production by THP-1 (A), PBMCs (B), and MDMs (C) after coincubation with trophozoites of A. castellanii (trophozoites) or stimulation with aCM. A total of 2 × 10^5^ of either target cells were challenged with either 2 × 10^4^ trophozoites, ratio of 10:1, or with 800 μl of aCM (final dilution of 4:5 in the culture medium). IL-8 concentration in THP-1 experimental supernatants was evaluated at 1.5, 4, 6, and 23 h poststimulation, whereas in both PBMCs and MDMs experimental supernatants it was evaluated at 1.5, 3, 5, and 18 h poststimulation. All three cellular models were stimulated with 10 ng/ml LPS as a positive control, whereas unstimulated cells (control) were considered the negative control. Experiments were performed in triplicate and repeated at least 3 times. Results represent the mean values ± SEM of either *n* = 5 (THP-1) or *n* = 3 (PBMCs and MDMs) samples. One-way ANOVA was applied for each time points. Dunnett's multiple-comparison test was performed to evaluate differences between the experimental condition means and the control at each time point. In the graphs, significances between the experimental conditions and the control are indicated as *P* values of <0.05 (*), <0.005 (**), and <0.0001 (***). Student *t* test was also applied at each time point to verify the significance of single pairs of means (single experimental condition versus control). In the graphs, significances between the experimental condition and the control, obtained with Student *t* test, are indicated as a *P* value of <0.0001 (***). On the right, the same results are expressed as fold change (FC) over the unstimulated cells.

IL-8 production by THP-1 cells was rapidly induced after coincubation with Acanthamoeba trophozoites, reaching the highest value at 4 h postincubation (920.7 ± 10.04, *n* = 5, *P* < 0.0001) and rapidly declining after, similar to the release pattern of TNF-α under the same experimental conditions. In contrast, aCM-induced IL-8 production by THP-1 was lower, reaching its highest values at 23 h poststimulation (213.2 ± 3.424 pg/ml, *n* = 5, *P* < 0.005) (Fig. 4A).

IL-8 production by human PBMCs was induced by both Acanthamoeba trophozoites and aCM from the earliest time point (1.5 h) and reached its highest values at 5 h poststimulation (Fig. 4B).

In human MDMs, both coincubation with trophozoites and aCM stimulation caused an immediate decrease of IL-8 concentration compared to control MDM cultures. Specifically, at 1.5 h postinfection the following values were detected: 201.3 ± 6.387 pg/ml, *n* = 3; 339.1 ± 7.734 pg/ml, *n* = 3; and 424.1 ± 17.95 pg/ml, *n* = 3 (Fig. 4C). The inhibitory effect induced by trophozoites was observed throughout the time course, whereas aCM induced a transient inhibitory effect, since IL-8 production increased by significant values between 5 and 18 h poststimulation (Fig. 4C).

LPS stimulation induced a significant release of IL-8 by THP-1 and PBMCs from the earliest time point (1.5 h), reaching the highest values at 5 h poststimulation, whereas in MDMs it caused a significant decrease of IL-8 concentration at 1.5 h, followed by a significant increase that reached a maximum peak at 5 h poststimulation (Fig. 4).

### A. castellanii (genotype T4) stimulated the production of anti-inflammatory cytokine IL-10 in human THP-1 cells, monocytes, and macrophages in a time-dependent manner.

**FIG 5:**
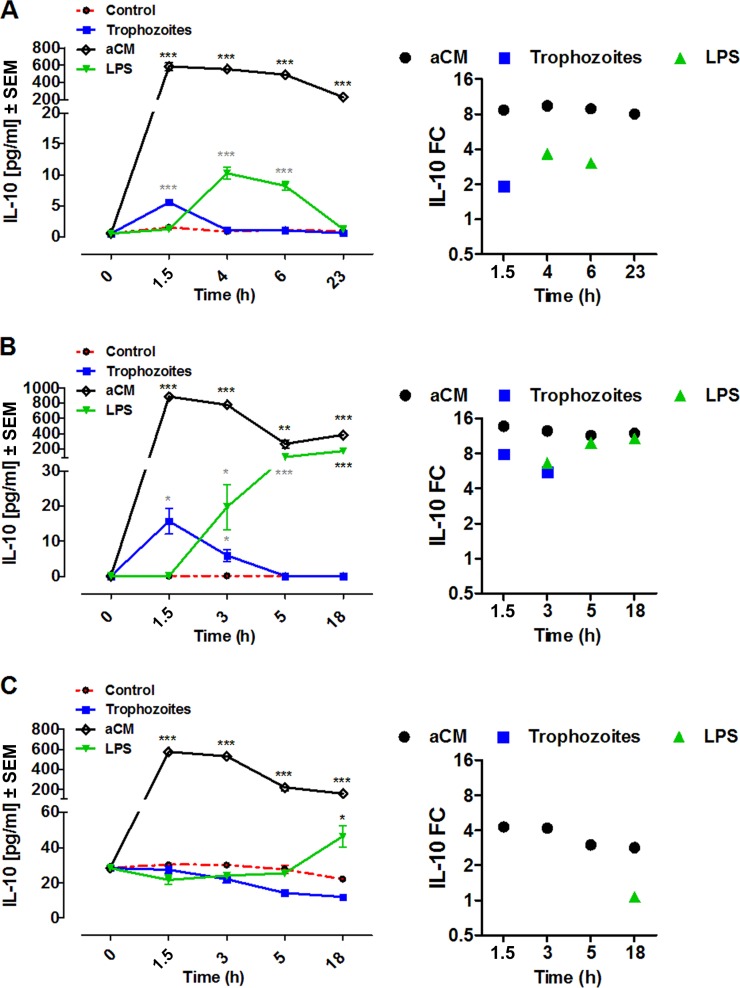
IL-10 production by THP-1 (A), PBMCs (B), and MDMs (C) after coincubation with trophozoites of A. castellanii (trophozoites) or stimulation with aCM. A total of 2 × 10^5^ of either target cells were challenged with either 2 × 10^4^ trophozoites, ratio of 10:1, or with 800 μl of aCM (final dilution of 4:5 in the culture medium). IL-10 concentration in THP-1 experimental supernatants was evaluated at 1.5, 4, 6, and 23 h poststimulation, whereas in both PBMCs and MDMs experimental supernatants it was evaluated at 1.5, 3, 5, and 18 h poststimulation. All three cellular models were stimulated with 10 ng/ml LPS as a positive control, whereas unstimulated cells (control) were considered the negative control. Experiments were performed in triplicate and repeated at least 3 times. Results represent the mean values ± SEM of either *n* = 5 (THP-1) or *n* = 3 (PBMCs and MDMs) samples. One-way ANOVA was applied for each time points. Dunnett's multiple-comparison test was performed to evaluate differences between the experimental condition means and the control at each time point. In the graphs, significance between the experimental conditions and the control are indicated as *P* values of <0.05 (*), <0.005 (**), and <0.0001 (***). Student *t* test was also applied at each time point to verify the significance of single pairs of means (single experimental condition versus control). In the graphs, significance between the experimental condition and the control, obtained with Student *t* test, are indicated as *P* values of <0.05 (*) and <0.0001 (***). On the right, the same results are expressed as fold change (FC) over the unstimulated cells.

IL-10 production by all three cellular models was significantly induced by aCM compared with the control. Coincubation with Acanthamoeba trophozoites also produced a significant release of IL-10 in THP-1 cells and PBMCs. IL-10 levels in supernatants from unstimulated THP-1 cells and MDMs ranged between 0.82 and 1.47 pg/ml and between 22.02 and 30.15 pg/ml, respectively. IL-10 was not detectable in nontreated PBMCs cultures at any time (Fig. 5).

IL-10 production by all three cellular models following aCM stimulation showed a similar pattern, characterized by a peak at 1.5 h poststimulation (THP-1 cells, 584.0 ± 46.58 pg/ml, *n* = 5, *P* < 0.0001; PBMCs, 883.30 ± 23.69 pg/ml, *n* = 3, *P* < 0.0001; MDMs, 574.4 ± 15.07 pg/ml, *n* = 3, *P* < 0.0001), a plateau (up to 3 to 4 h), and a slow decline, maintaining significant values throughout the course of stimulation. The infection with Acanthamoeba trophozoites evoked a transient increase of IL-10 concentration by THP-1 cells that was significant only at 1.5 h postinfection (*P* value of <0.0001 by Student *t* test), whereas in PBMCs it was significant at both 1.5 and 3 h poststimulation (*P* value of <0.05 by Student *t* test) (Fig. 5A and B).

In all three cellular models examined, LPS stimulation induced a delayed and gradual IL-10 production compared to Acanthamoeba stimulation (Fig. 5).

**FIG 6:**
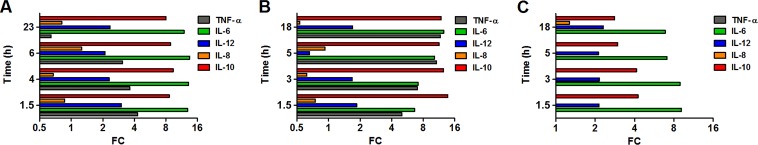
Schematic summary of the cytokines released by THP-1 (A), human PBMCs (B), and human MDMs (C) after stimulation with aCM. Incubation with aCM induced the release of a greater number of cytokines. In particular, IL-10, IL-6, and IL-12 were immediately produced, by both human monocytes (THP-1 cells and PBMCs) and human macrophages, and detected throughout the period of stimulation. Their increase was constant overall during the course of observations. aCM induced TNF-α production only by monocytes, although showing a different release pattern: its concentration increased in PBMCs during the time course (FC of 4.69 to 11.69), whereas in THP-1 cells it decreased (FC of 4.29 to 0.64). aCM-induced IL-8 production increased (FC of 1) at 5 to 6 h poststimulation (THP-1 and PBMCs) or at 18 h poststimulation (MDMs).

**FIG 7:**
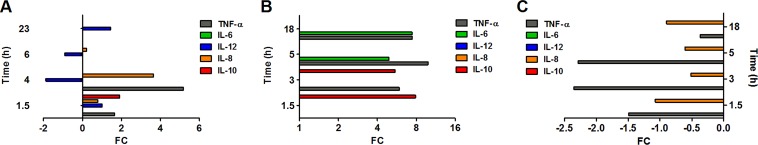
Schematic summary of the cytokines released by THP-1 cells (A), human PBMCs (B), and human MDMs (C) after coincubation with Acanthamoeba trophozoites. During coincubation with trophozoites of Acanthamoeba, human monocytes (THP-1 cells and PBMCs) produced IL-10 from the earliest time points only for a short period of time; subsequently it produced TNF-α and IL-8. In contrast, trophozoites induced a particular decrease of IL-8 and TNF-α in human macrophages.

Cytokine and chemokine release patterns induced by either aCM or trophozoites in all three cellular models, presented as fold change (FC) over unstimulated cells, are summarized in Fig. 6 and 7, respectively.

## DISCUSSION

*In vivo* and *in vitro* studies suggest that both humoral and cell-mediated adaptive immune responses are induced by Acanthamoeba, although they are not efficient in resolving the infection (32 – 35). Consequently, it is the general consensus that the innate immune response plays the main role in preventing the establishment of Acanthamoeba infections and is responsible for the clearance of the amoebae from host tissues (16, 36). Little is known about the interactions between Acanthamoeba and monocytes/macrophages in *in vitro* human models and, in particular, the pattern of proinflammatory and anti-inflammatory cytokines that both whole trophozoites and amoeba-released products induce.

Our study indicates that human monocytes and human macrophages can recognize and respond to Acanthamoeba trophozoites as well as to amoebic soluble compounds, releasing cytokines and chemokines with different kinetics and intensity. The present findings, therefore, suggest that amoeba adhesion to these cells is not a prerequisite to induce their response to Acanthamoeba.

In particular, our data show that whole trophozoites and amoebic products are capable of inducing a different response by the target cell types, with the latter being more effective in stimulating all cytokines (TNF-α, IL-6, IL-12, and IL-10) and the chemokine (IL-8) investigated. The effect of the trophozoites appears to be more complex, since they can both stimulate and inhibit cytokine production. Indeed, they induce IL-10, IL-6 (only by PBMCs), TNF-α, and IL-8 release in monocytes, but they inhibit the production of TNF-α and IL-8 by macrophages. Trophozoites do not exert any effect on the production of IL-12 by PBMCs and MDMs, while in THP-1 cells they modulate its release both in a positive and negative way.

In our opinion, the different cytokine patterns induced by either trophozoites or aCM may be due, in part, to the actual composition of the aCM. In accordance with our previous studies (22), aCM contains the released products of a number of trophozoites at 160 times higher than the number of trophozoites actually used for the trophozoite/target cell coincubation models. In fact, it was not possible to use trophozoite/target cell ratios greater than 1:10, since a higher number of trophozoites induced lower cytokine production and caused a cytopathic effect. Nevertheless, our data do not preclude the possibility that these differences are specific to products secreted by trophozoites.

The response of monocytes and macrophages to Acanthamoeba showed differences, probably due to a different expression of receptors and coreceptors following cell differentiation. In this regard, we exclude the significant interference of individual factors, as the data obtained by monocytes and macrophages isolated from different blood donors showed a remarkable homogeneity. Moreover, the results obtained from the characterization of monocytes and macrophages before each experiment indicate that cell populations used in our experiments were homogeneous. We suggest that the responsiveness of target cells varied according to the following order: PBMCs > THP-1 > MDMs.

THP-1 cells are a human leukemia monocytic cell line extensively used to study the modulation of monocytes and macrophages during infectious conditions without using human blood samples (37). THP-1 cells in the monocyte state can be differentiated into a macrophage-like phenotype using either phorbol-12-myristate-13-acetate (PMA) or 1α,25 dihydroxyvitamin D3 (vD3) (38, 39). Our data indicate that THP-1 cells are a good experimental model for the study of Acanthamoeba interactions with human monocyte/macrophage. Interestingly, although our experiments have been conducted on THP-1 cells not subjected to any previous activation, we found that their behavior regarding the release of IL-6 was more similar to that of MDMs than PBMCs. Indeed, the dynamics and profile of aCM-induced IL-6 production by THP-1 cells and MDMs were quite dissimilar from those induced by LPS stimulation. Moreover, aCM caused significantly more rapid IL-6 production than stimulation with LPS. However, in PBMCs, following aCM stimulation, IL-6 release showed a trend similar to that obtained by LPS stimulation. In addition, Acanthamoeba trophozoites failed to induce IL-6 production in both THP-1 cells and MDMs. This diversity observed in the responsiveness of PBMCs and THP-1 cells lead us to hypothesize that the latter are differentiating from a monocytic toward a macrophagic stage.

IL-10 is now considered a key regulator of innate immunity, and it is known to suppress inflammation and macrophage activity by inhibiting the production of gamma interferon (IFN-γ), IL-2, IL-12, IL-18, and TNF-α, as well as other cytokines (18, 40, 41); nevertheless, the molecular mechanisms underlying the signaling events that lead to IL-10 anti-inflammatory actions are not entirely clear. To our knowledge, this is the first report demonstrating that Acanthamoeba trophozoites can induce the production of IL-10, a potent immunosuppressive and anti-inflammatory cytokine, by human mononuclear cells *in vitro*. Similarly, aCM can also induce IL-10 production, although showing greater concentration than that with trophozoites. Specifically, we observed that Acanthamoeba induced an early production of IL-10 by both human monocytes (THP-1 cells and PBMCs) and MDMs, reaching its peak after just 1.5 h poststimulation and maintaining high levels up to 18 h postinfection. These data are very important, since the early phase of infection is the key step where an inflammatory, regulatory, and/or memory response is preferentially induced. In terms of timing, this is the moment in which IL-10 can radically influence subsequent responses (42, 43). Indeed, it has been demonstrated that the early production of IL-10 can lead to a premature change from cell-mediated immunity to humoral adaptive responses, causing persistent or chronic infection (44). We hypothesize that the release of IL-10 is due to the activity of a soluble molecule(s) secreted by Acanthamoeba. At the moment, the chemical nature of this molecule(s) has not been characterized; however, studies for its identification and characterization have already being designed. Numerous recent studies have demonstrated that IL-10 is triggered by Leishmania spp., Plasmodium spp., Toxoplasma gondii, and Trypanosoma spp. However, the specific protozoan components and the mechanisms involved in this immune event are still unclear (45).

Our previous study on the effects caused by Acanthamoeba-released products on THP-1 cells has shown that coincubation with aCM induces production of IL-6, while heat treatment (95°C for 10 min) and aCM fractionation (using Centriprep-30 and Centriprep-10 microconcentrators with 30- and 10-kDa cutoffs, respectively) abolishes its activity (29). These data were confirmed and broadened by our present study, where we showed the ability of aCM to induce remarkable levels of IL-6, which reaches its highest peak much earlier in THP-1 cells and MDMs than in PBMCs. At the moment, we cannot establish whether IL-6 release is induced directly by amoebic metabolite stimulation or indirectly by the autocrine action of IL-10 (considering its increasing concentration in the culture medium), or again if it depends on both these causes. In fact, it is known that IL-10 can moderate infection-associated immune pathology linked with strong Th1 responses (46). The suppressive activity of IL-10 toward Th1 responses enables the development of Th2 responses characterized by the production of IL-6 (18, 41). IL-6 acts during early and late innate immune events; indeed, it damps the early innate immune responses by inducing neutrophil apoptosis and the production of IL-1 and TNF-α antagonists, initiating the late innate immune events, such as the recruitment of monocytes and their differentiation into macrophages. Subsequently, IL-6 promotes antibody production by B lymphocytes. Although this effect on humoral effector mechanisms related to B lymphocytes is important in the resolution of some helminthic and protozoan infections, it is ineffective in resolving other infections, such as those caused by Plasmodium and Acanthamoeba (47, 48).

It is known that TNF-α is an early cytokine rapidly released by monocytes and macrophages to activate neutrophils and endothelial cells (49). It has also been shown that Acanthamoeba spp. are not susceptible to killing by TNF-α and that this cytolytic factor, which promptly induces trophozoite encystment, may paradoxically offer protection from macrophage phagocytosis (17). A. castellanii trophozoites and aCM induced various levels of TNF-α in human monocytes (THP-1 cells and PBMCs), but despite the ability to produce this potent proinflammatory cytokine being included in the antimicrobial arsenal of macrophages, both trophozoites and aCM failed to induce TNF-α in human macrophages. However, treatment with LPS caused a significant release of TNF-α by human macrophages, indicating that, under our experimental conditions, they had the potential to produce this cytokine. Furthermore, the aCM-mediated TNF-α production observed in monocytes confirms our previous findings obtained in THP-1 cells (29). Interestingly, comparing the release curves of TNF-α and IL-10 in THP-1 cells and PBMCs after stimulation with either LPS or Acanthamoeba, it is clear that LPS causes early production of TNF-α but delayed IL-10 production, underlying a correlation between the two cytokines: as the latter increases, the concentration of TNF-α decreases. Interestingly, both Acanthamoeba trophozoites and aCM stimulate a peculiar early release of both cytokines, but in this case we also can observe a clear correlation between the increase of IL-10 and decrease of TNF-α.

IL-12 is a proinflammatory cytokine that induces phagocyte cytotoxic activity, the production of reactive nitrogen species (RNS) and reactive oxygen species (ROS), and the stimulation of IFN-γ by natural killer (NK) cells, cytotoxic T lymphocyte (CTL) development, and/or Thl lymphocyte responses (50). We also observed that aCM, in contrast to trophozoites, causes IL-12 release in human THP-1 cells, PBMCs, and MDMs for the whole duration of the observations, albeit of low intensity.

Monocytes/macrophages recruit neutrophils and other granulocytes to the site of infection through the release of IL-8 that, in target cells, induces a series of physiological responses required for migration and phagocytosis, such as an increase in intracellular Ca^2+^, exocytosis, and the respiratory burst (51). *In vivo* studies using animal models have demonstrated that neutrophils are recruited following corneal trauma associated with Acanthamoeba infections and that they are present in granulomas in the brain, preventing the spread of the protozoan (14, 16, 52). In this context, we found that Acanthamoeba can indirectly modulate the recruitment and activity of neutrophils, since both trophozoites and aCM stimulate the production of IL-8 by human monocytes, although of low intensity. On the other hand, only aCM induces the release of IL-8 by human macrophages, whereas trophozoites cause a significant decrease of this chemokine compared to noninfected cultures.

Taken together, our findings indicate that challenge with Acanthamoeba or their soluble products can upregulate both proinflammatory cytokines (TNF-α, IL-6, and IL-12) and anti-inflammatory cytokine (IL-10), suggesting that this protozoan infection results from the early induction of the concurrent conflicting immune responses. Human monocytes, compared to monocyte-derived macrophages, have a greater responsiveness toward these amoebae, especially toward trophozoites, by releasing both cytokines and the chemokine IL-8. Therefore, we suggest that monocytes are involved in preventing the hematogenous spread of Acanthamoeba trophozoites rather than directly acting at sites of infection.

In conclusion, results reported in this paper confirm that the production of proinflammatory cytokines and chemokines by monocytes and macrophages can play a role in the inflammatory response during Acanthamoeba infections. Moreover, they demonstrate for the first time that Acanthamoeba stimulates the production of IL-10 in human innate immune cells. This anti-inflammatory cytokine might play a role in both promoting the immune evasion of Acanthamoeba and limiting the induced inflammatory response.

Further *in vitro* studies are in progress to identify the nature of amoebic surface molecules and of the secreted products which stimulate cytokine and chemokine release by monocytes/macrophages, as well as to evaluate the role of innate immune receptors and/or coreceptors in the interactions that occur between pathogenic Acanthamoeba and mononuclear immune cells. Understanding the immunological mechanisms involved in host resistance will hopefully provide useful new insights for the development of effective pharmaceutical treatments for Acanthamoeba infections.
